# Development of cellulose acetate poly acrylonitrile (CAPA)–SiC/epoxy coating to mitigate corrosion of copper in chloride containing solutions

**DOI:** 10.1038/s41598-024-70166-3

**Published:** 2024-09-09

**Authors:** Nehal Bargout, Abd El-Hady B. Kashyout, Magdy A. M. Ibrahim, Ahmed El Nemr

**Affiliations:** 1 Environmental Division, National Institute of Oceanography and Fisheries (NIOF), Kayet Bey, Elanfoushy, Alexandria Egypt; 2 Electronic Materials Research Department Advanced Technology and New Materials Research Institute, City of Scientific Research and Technological Applications (SRTA-City), New Borg El-Arab City, P.O. Box 21934, Alexandria, Egypt; 3 Department of Chemistry, Faculty of Science, Ain Shams University, Cairo, Egypt

**Keywords:** CAPA–SiC nanocomposites, Marine sponge, Cellulose acetate poly acrylonitrile, Corrosion inhibitor, Copper corrosion resistance, Epoxy coating, Electrochemistry, Corrosion

## Abstract

A new conducting polymer of the cellulose acetate poly acrylonitrile (CAPA)–SiC composite was produced using an in situ oxidative polymerization technique in an aqueous medium. SiC was synthesized from *Cinachyrella sp.* as a source of carbon and silicon at 1200 °C under an argon atmosphere via a catalytic reduction process. The structure and morphology of the CAPA–SiC composite were characterized using surface area studies (BET), X-ray diffraction (XRD), Fourier transformation infrared spectroscopy (FT-IR), and surface morphology (SEM & TEM). To protect copper, the produced CAPA–SiC composite was mixed with commercial epoxy paint using a casting technique, and the copper surface was coated with the three components of the CAPA–SiC/epoxy paint mixture. The corrosion inhibition improvement of the CAPA–SiC/paint coating was assessed using electrochemical impedance spectroscopy followed by Tafel polarization measurements in a 3.5 wt% NaCl solution. The corrosion protection ability of the CAPA–SiC/epoxy coating was found to be outstanding at 97.4% when compared to that of a CAPA/paint coating. SEM and XRD were used to illustrate the coating on the copper surface.

## Introduction

Copper is believed to be the first metal discovered and utilized by human beings. It is commonly used in various industries, including electrical, lighting, machinery, fabrication, building, and military. Additionally, it is extensively employed in marine environments for important purposes such as heat exchangers and pipelines^1–3^. Significant pains must be made to avoid copper deterioration and to guarantee that copper apparatus performs efficiently throughout its life. Applying certain settings, copper can be susceptible to severe deterioration, particularly in extremely corrosive environments^4–7^ such as industrial and marine fields that contain O_2_, Cl^−^, and Cl_2_. Among these, oxygen and chlorine are essential components of the seawater environment causing damage to metals through the active corrosion process^8^. Chlorine is a very abundant element in seawater salinity with a concentration of about 3.5%, thus atmospheric oxygen tends to strongly liquefy into seawater. Consequently, the marine environment suffers from very severe corrosion conditions for metal structures. These aggressive conditions lead to thinking about the physical or chemical barrier that impedes the aggressive Cl^−^ and O_2_ from contact with metal surfaces^9–13^. Flexibility, high hardness, and convenient adhesion are the control characteristics of roofless coating as an outer layer on metal that is considered a structural adaptation to mechanical abuse stress, swell, or climate conditions^14–18^.

Plenty of attempts^19–23^ have been made to preserve copper against corrosion, the most significant of which is coating^24,25^ and inhibitor technologies^26–28^. Coating efficiently protects the metal against corrosion induced by environmental factors. Typically, the coating alone can adequately protect the metal with good and acceptable percent without any addition of corrosion inhibitor. Combining different corrosion protection strategies, such as coatings and inhibitors, can change a coating’s properties and greatly increase its protective effect. However, corrosion inhibitors can also negatively affect a coating’s strength, uniformity, and corrosion resistance, and the corrosion-resistant agent itself may fail as a result of leakage from the coating’s surface^29^.

Another important factor that must be in our interest before choosing the method of corrosion treatment, is the type of system that suffers from corrosion. Marine industries and buildings such as gas and oil pipelines suffer from external corrosion, so it is preferable to use paint because it is not reasonable to put an inhibitor in seawater or oceans^30^. To improve the coating quality, inorganic and organic inhibitors instead may be included within the coating structure especially, polymers such as cellulose acetate and poly acrylonitrile have been reported for corrosion hindrance. Cellulose acetate has been broadly studied for its intermediate cost and easy processability^31–40^. Also, polyacrylonitrile has been directly studied for its inhibition effect on metal corrosion^31,41,42^. The presence of groups that include alkoxy, alkyl, amine, or imine on the benzene ring will motivate both corrosion resistance and physical properties of cellulose acetate or polyacrylonitrile. These groups also may lead to steric hindrance as well as p-electron effects^43^.

Consequently, we created a (CAPA–SiC) composite in this study by copolymerizing cellulose acetate and polyacrylonitrile with SiC acting as a dopant and cerium ion acting as a free radical. Additionally, we prepared (CAPA–SiC)/hard top XP paint materials using this composite and applied them to a copper surface. The (CAPA–SiC) composite’s arrangement, thermal stability, and electrochemical measurements were examined and evaluated. With a 3.5 wt% NaCl solution acting as the corrosive medium, the corrosion resistance of the (CAPA–SiC) composite/paint coat was examined using Tafel polarization and electrochemical impedance spectroscopy. In-depth discussions were also had regarding the corrosion protection mechanism and the coat’s surface analysis.

## Material, methods, and characterization

Commercial copper samples of the following chemical composition in mass percent: 59.3 Cu, 38.7 Zn, 0.8 Fe, 0.1 Mo, 0.2 Sn, 0.9, Ca and traces of other elements have been used as the working electrode. The working electrode used was designed into a cylindrical shape of copper was fixed into a polytetrafluoroethylene (PTFE, Teflon) rod with an epoxy resin in such a way that no crevice was created, and only one side of a constant surface area (1.281 cm^2^) was exposed to the solution. A thick copper wire was screwed to the metal specimen and introduced into an insulated glass tube for electrical connection copper. Before using the samples for the relevant experiment, they were completely rinsed with distilled water, degreased with ethanol, rinsed with acetone, and dried in the air. For weight loss and immersion test, the same type of copper specimens of cylindrical shape without epoxy resin.

### Synthesis materials

Cellulose acetate from ALPHA CHEMIKA, and ammonium cerium (IV) nitrate FROM Fisher Scientific UK. ACRYLONITELE 99% from LOBA CHEME (APS, CASRN: 7727-54-0) and nitric Acid (69–72%) from ADVENT and sodium chloride extra pure from Oxford lab chem.

### Synthesis of SiC

The scientific classification of *Cinachyrella* sp.’s marine sponge (MS) Egyptian seawater in the Abukir region of the Mediterranean starts with Kingdom: Animalia and finished with Genus: *Cinachyrella* that was used to make the specimen. Before utilizing the marine sponge, MS underwent washing, evaporation, and grinding using a millpulveristic 2. To create a jelly-like slurry, only 4.0 g of MS and 1.0 g of prepared metals-catalysts were combined with deionized H_2_O in a bath of ultrasonic waves for 1 h. To turn the slurry into powder, it was baked for 75 h at 80 °C to evaporate the water.

The resultant precipitate from the preceding phase was hosted in a tube furnace (Tubular Furnace Nabertherm B180 (RT 50/250/13)) of alumina cylinder at temperatures between 1200 °C for 4 h, monitored by the gradual cooling rate of five °C/min. The last yields were soaked in 1 M NaOH at 50 °C to remove any amorphous silicon dioxide impurities. Also, another soaking step in 5% HF for 6 h to obtain a satisfactory yield of SiC.

### Synthesis of CAPA–SiC composite

*Cinachyrella* sp. was used as the starting material for the fabrication of SiC–SiO_2_ composite via catalytic reduction under an argon atmosphere at 1000 °C. Before pyrolysis, the sponge had a pale yellow color, then it was transformed to a shiny black, brittle product but, after soaking in concentrated 5% HF solution at room temperature, the brittleness decreased and blacker color appeared. CAPA–SiC composite was created by free radical polymerization under argon using a cerium ion redox scheme as an initiator. Only 5 g of cellulose acetate (CA) and 0.3 g of SiC were liquefied in 60 mL double distilled water in a three-necked flask with rotation at high speed using magnetic stirring for 3 h under argon to form the first reaction solution. Only 0.5 g of cerium salt was dissolved in 1 M nitric acid for 30 min. as the second reaction solution. The second solution was added to the first solution for free radical polymerization at 60 °C for 8 h. The designed polymer was filtrated and tracked several times by washing with distilled water and finally dried in an oven at 50 °C. The product gets ready to be inducted into the commercial paint.

### Synthesis of CAPA–SiC composite coating on copper

Commercial copper samples of the following chemical composition in a mass percent: 59.3 Cu, 38.7 Zn, 0.8 Fe, 0.1 Mo, 0.2 Sn, 0.9 Ca, and traces of other elements have been used as the working electrode. Copper samples were cut into 0.5 × 1 cm measurements and their outsides were refined manually using emery papers up to 1200 followed by washing with acetone (Sigma-Aldrich, USA), ethanol liquids (Oxford, India), and then aeration at room temperature. To prepare coatings, CAPA–SiC composite powder was mixed with UN 1866 resin solution and UN 1863 hard top XP paint in calculated amounts of 0.3 g, 5 mL, and 20 mL, respectively. To complete dispersion, magnetic stirring was used for about 45 min. Only 0.25 mL of the prepared mixture for coating was cast on copper. Copper/paint coating was still in the air at room temperature for 72 h for complete drying. The samples are ready for corrosion measurements.

### Characterization

Via (BELSORP-Mini II, BEL Japan, Inc.) at 77 K and a relative pressure (P/P°) range of 0.001–1, both pore size and surface area of the CAPA–SiC composite were studied^44^. The coating polymer’s specific surface area (SBET), total pore volume (VT), and mean pore diameter (DP) were determined using the Brunauer–Emmett–Teller (BET) method^45–47^. As per the BELSORP analysis program software^48–54^, the CAPA–SiC composite’s micropore surface area (Smi), micropore volume (Vmi), mesopore surface area (Sme), and mesopore volume (Vme) were ascertained through the t-plot and Barrett–Joyner–Halenda (BJH) methods^38,39^.

The structure morphology and crystallographic arrangement of the CAPA–SiC composite were achieved using Scanning Electron Microscopy (SEM, (JEOL-JSM-5300LV, Tokyo, Japan)) and XRD investigation on the treated copper before and after sodium chloride performance using an X-ray diffractometer (D2 BRUCKER) with a Cu target and nickel filter through Cu Kα energy of λ = 0.150598 Å, correspondingly^55–57^.

### Corrosion investigation and characterization

#### Weight loss measurements

The mass loss experiment was conducted in a 500 mL glass cup filled with a 3.5 wt% sodium chloride solution, which was temperature-controlled using a sensor. The copper samples used for the weight loss analysis had a rectangular shape and a total exposed area of 2.5 cm^2^. Before the coating process, the blank samples were smoothed using gradual emery paper, weighed, and suspended in the 500 mL 3.5 wt% NaCl solution. The blank copper samples were also smoothed using gradual emery paper, washed with water, and then with ethanol before starting the immersion test. After each month of the 4-month immersion test at a temperature of 25 °C, the samples were removed, cleaned with distilled water, and then weighed. To ensure accuracy, measurements of weight loss were conducted only twice due to the long period of copper immersion that was necessary to yield noticeable weight loss. Finally, the average weight loss result was reported^58–61^.

#### Electrochemical measurements

Three electrodes were used in the electrolytic cell designed for the electrochemical measurements: (a) a saturated calomel electrode (SCE) was used as the reference electrode; (b) a Pt sheet was used as the auxiliary electrode; and (c) the copper surface of the working electrode was either uncoated or coated with CAPA–SiC composite paint. Before making any measurements, the uncoated copper surface was thoroughly cleaned and polished, but the coated copper samples were thoroughly cleaned only to keep the coating layers without any change. Every experiment was run in a 3.5% NaCl solution at 25 °C. For runs and analysis, Metrohm Autolab PGSTAT30 2N software version 1.11.2 from Nova was utilized^62–64^.

Utilizing a potential ranging from -250 to + 250 mV (SCE), Tafel’s studies on the impact of CAPA–SiC composite/paint coatings on copper help assess current density (*i*_corr_). By extrapolating the cathodic and anodic Tafel lines, the electrochemical parameters, corrosion current density (*i*_corr_), and corrosion potential (*E*_corr_), are determined^65^. Moreover the surface coverage (*θ*) and inhibition efficiency (*IE*%) and had been equated using (1, 2):

1 $${\text{\% }}IE = ((i_{corr} - i_{inh} )/i_{corr} ) \times 100$$

2 $$\uptheta = \frac{{{\text{\% }}IE }}{100}$$

where the (*i*_corr_) is the corrosion current density of non-coated samples and (*i*_inh_) is the corrosion current density of CAPA–SiC composite/paint coat on copper substrate.

The EIS test is a type of non-destructive electrochemical test that was performed using AC signals at open circuit potential and frequency ranges from 0.1 Hz up to 100 kHz with an amplitude of five mV peak to peak. A selected fitting model of impedance data was used to calculate Cdl (double-layer capacitance), *R*_ct_ (charge transfer resistance).

Equation (3) was used to calculate *IE*% (inhibition efficiency), and Eq. (2) was used to *θ* (surface coverage degree from impedance data), which were the fundamental parameters obtained from EIS measurements^66^:

3 $${\text{\% }} IE = ((R_{{CT_{inh} }} - R_{{CT_{corr} }} )/R_{{CT_{inh} }} ) \times 100$$

where the charge transfer resistances of uncoated copper and coated copper with CAPA–SiC composite/paint are symbolized by *R*_CTcorr_, and *R*_CTinh_, respectively.

## Results and discussion

**Figure 1:**
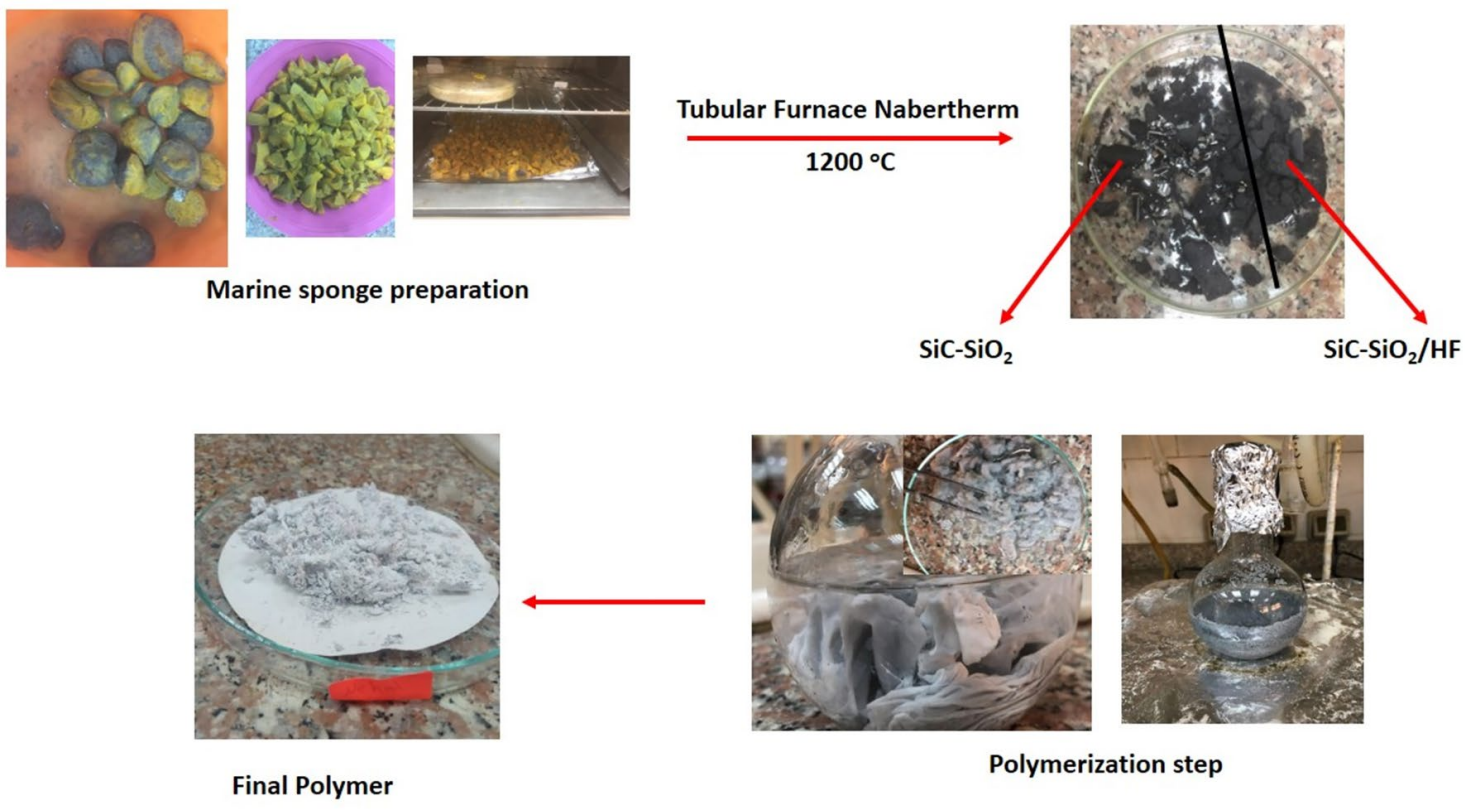
Laboratory preparation of CAPA–SiC composite synthesis.

The final product was injected through the polymerization step giving a grey polymer with a clear surrounding solvent (Fig. 1).

### FTIR characterization

**Figure 2:**
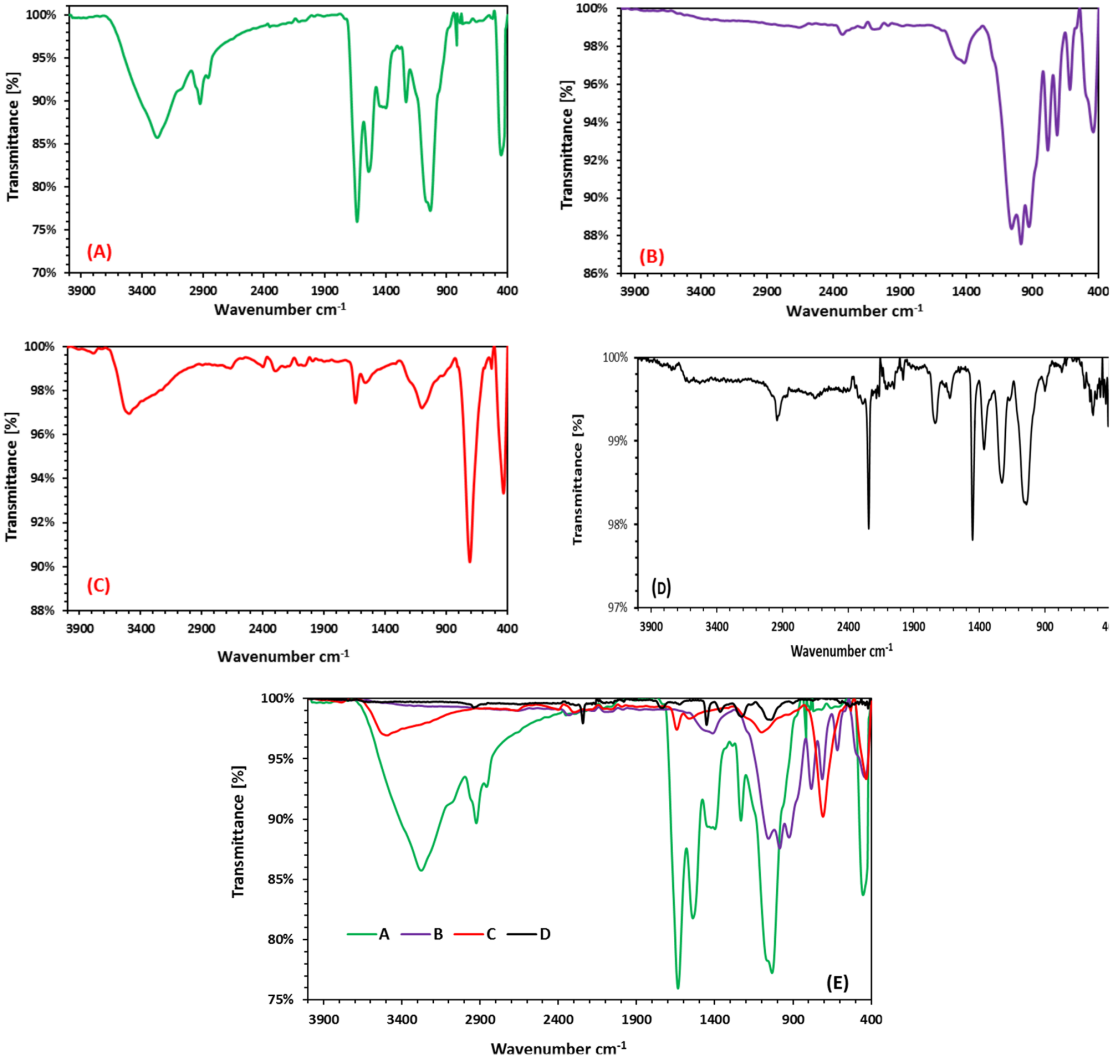
FT-IR spectra of (**A**) MS, (**B**) SiC–SiO_2_ nanocomposite, (**C**) SiC–SiO_2_ nanocomposite after soaking in HF, (**D**) CAPA–SiC composite, (**E**) Group of the spectra A, B, C and D.

FTIR spectra of starting material and resulting copolymer were obtainable in Fig. 2. The typical peaks of marine sponge stayed 3274, 2927, 1637, 1535, 1035, 815 and 449 cm^−1^, which resulted from the stretching vibration of O–H, C–H, C=O, C–C, Si–O, Si–C and Si–O^67^ respectively (Fig. 2A). All the above peaks disappeared except the last 3 peaks by pyrolysis of marine sponge at 1200 °C in argon atmosphere followed by appearance new peaks at 711 of Si–Si, decrease intensity of Si–O with compared to marine sponge, extra intensity and noticeable integration of Si–C (Fig. 2B). At the same positions for Si–C after soaking in HF, product had a representative absorbance at 815 cm^−1^, which was recognized to Si–C functional groups and acceptable decrease in Si–O peaks (Fig. 2C). Synthesis path for the CAPA–SiC composite copolymers was given in Fig. 1. CA is distinguished by frequent carbon atoms interconnected with OH groups, and the redox polymerization among CH–OH groups of CA powder and cerium ions (IV) in presence of SiC nanotubes consequences in the radical creation on carbon atoms of glucose of cellulose acetate. Subsequently the injection of PA monomers, the produced radicals were relocated from CA to the carbon double bonds in PA monomers, leading to the spread response between the PA monomers until the chain end. The created CAPA–SiC composite polymers were also grey powder. FTIR spectra of CAPA–SiC composite copolymers polymers were obtainable in Fig. 2D. The typical peaks of CA were 3471, 1737, 1640, 1369, 1267, 1062, 815 and 711 cm^−1^^68^, which resulted from the stretching vibration of hydroxyl, carbonyl, C=C, C=N, Si–O, Si–C and Si–Si, respectively^69^.

### BET characterization

**Figure 3:**
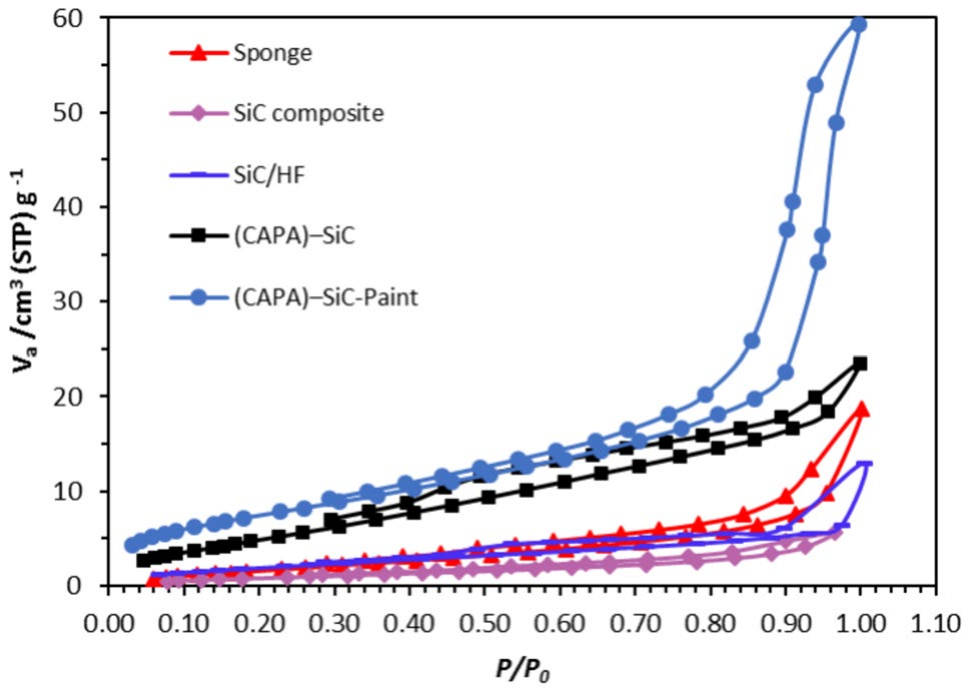
Isotherm with highlighted range for calculation of specific surface area (BET) of different materials.

**Figure 4:**
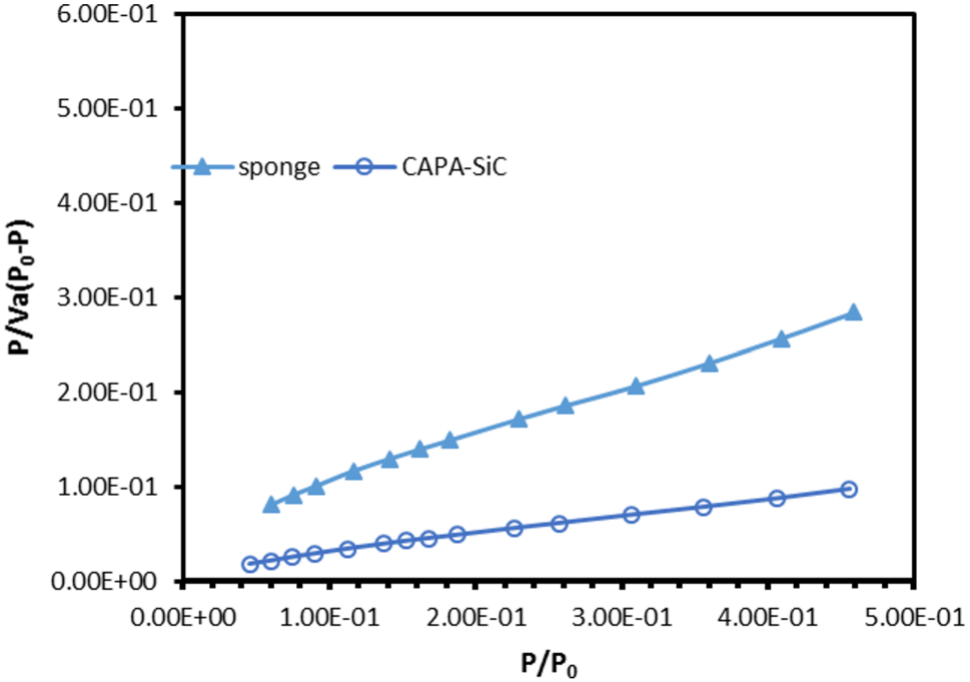
N_2_ adsorption–desorption isotherm of different materials is type IV following the IUPAC classification.

Nitrogen desorption and adsorption studies were directed to inspect both surface areas besides pore volume for copper surface coats in 3.5 wt% NaCl (marine sponge Fig. 3, SiC–SiO_2_ composite, Fig. 3, SiC–SiO_2_ composite after soaking in HF (Fig. 3), CAPA–SiC composite copolymers (Fig. 3), and CAPA–SiC composite copolymers mixed with epoxy paint, Fig. 3. From data that were listed in Table 1 and Fig. 3, we notice the variation of total pore volume decrease to be minimum in case of SiC composite with little increase after treatment by HF. The total pore volume of epoxy paint was affected by mixing with CAPA–SiC as a filler to be the minimum case. Figure 4 shows changes in N_2_ adsorption–desorption isotherm to type-V with significant hysteresis loops agreeing to the IUPAC classification^70^. This suggests that the pores are frequently mesoporous and macroporous. Perfectly, the BET surface area of CAPA–SiC composite copolymers mixed with epoxy paint is much higher than those of the other samples. The pore volume decreases through SiC samples but, it increases by doping SiC into a polymer chain and this is related to being SiC filler into the polymer.

**Table 1:** The porous structure factors of marine sponge SiC–SiO_2_ composite, SiC–SiO_2_ composite after soaking in HF, CAPA–SiC composite copolymers and CAPA–SiC composite copolymers mixed with epoxy paint.

Material	SA (m^2^/g)	*V*_m_ (Cm^3^/g)	Mean pore diameter (nm)	Total pore volume × 10^−2^
Marine sponge	8.0371	1.8466	12.767	2.5652
SiC–SiO_2_ composite	3.8005	0.8732	15.560	1.4784
SiC–SiO_2_ composite + HF	4.9014	1.8213	7.7196	1.5299
CAPA	12.59	2.8931	8.8352	2.7814
(CAPA)–SiC composite	21.68	4.9831	6.4180	3.4800
epoxy paint	28.034	6.4410	12.547	8.7938
(CAPA)–SiC composite + epoxy paint	24.787	5.6948	6.6629	4.1288

### XRD analysis of different coatings on the copper surface

**Figure 5:**
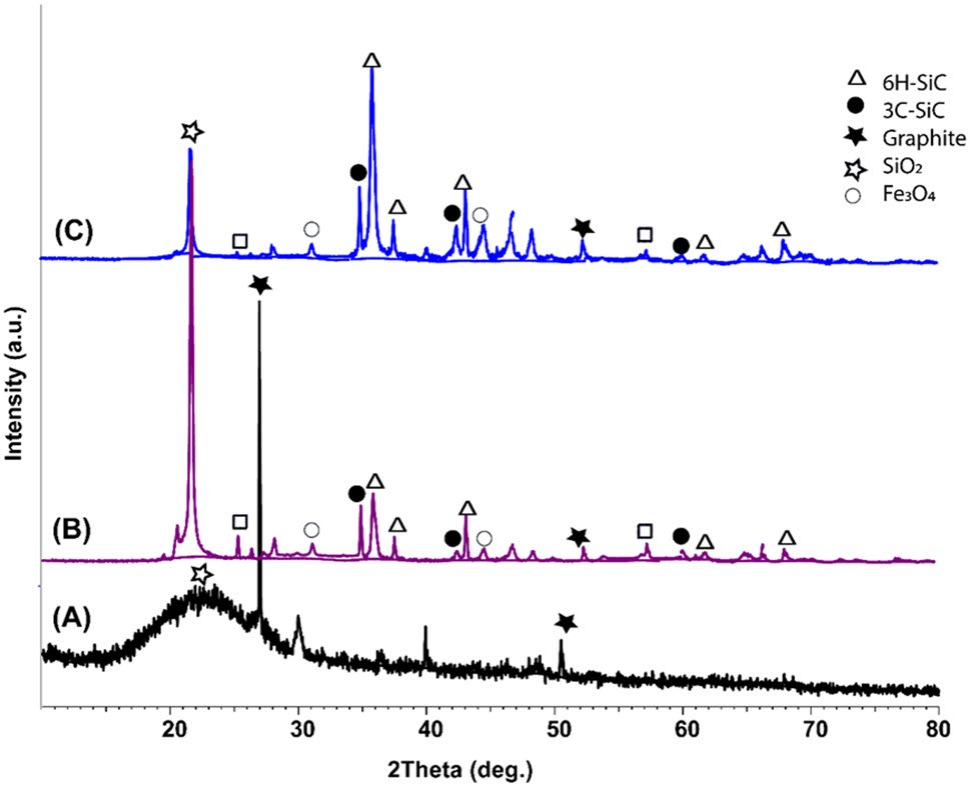
X-ray diffraction patterns after washing of (**A**) Marine sponge, (**B**) SiC–SiO_2_ composite, (**C**) SiC–SiO_2_ composite after soaking in concentrated HF.

The XRD pattern of marine sponge before the pyrolysis process showed a peak at 2*θ* = 27° signifying the (002) plane of the graphite with the compatible of (JCPDS Card no: 6-6212) equivalent to carbon content with amorphous SiO_2_ structure at 2*θ* = 22.5°, representing the attendance of (101) plane (JCPDS Card no. 01-086-1561)^71,72^. According to XRD results of marine sponges, the sponge is a suitable and essential starting component in SiC synthesis (Fig. 5A).

XRD data studies in Fig. 5B display that growing the reaction heat leads to an important attendance of both SiO_2_ crystalline, silicon carbide, and low peak strengths of CNT, silicon, and Fe_3_O_4_ catalyst nanoparticles. Each constituent in the resultant composite has its inhibition effect of copper coating^73–75^. Through increasing temperature up to 1000 °C, the amorphous SiO_2_ converted into a polycrystalline structure of a conforming peak positioned at 2*θ* = 21.5° of the (100) plane of polycrystalline SiO_2_ (JCPDS Card no: 29-1129)^76^.

Also, two phases of SiC of high intensities were noticed; the first was hexagonal structure 6H-SiC, and the second was cubic 3C-SiC. Peaks conforming to the presence of 6H-SiC were sensed at 2*θ* = 36°, 37°, 42°, 60° and 68° of planes (102), (103), (104), (110) and (202), correspondingly (JCPDS Card no: 29-1128). Peaks of 3C-SiC were detected at 2*θ* = 35.6°, 41.3°, 59.9°, and 72 of planes (111), (200), (220), and (311), respectively (JCPDS Card no: 75-0254). Also, weak strengths peaks of equally Fe_3_O_4_ that attributed to XRD peaks at 2θ of 18°, 30.1°, 35.5°, 43.1°, 57.0°, and 62.6° corresponding to diffraction planes (220), (311), (400), (511), (442), and (440), respectively (JCPDS card 19-0629) and CNT at 2*θ* of 25°, 43° and 56.8° matching to diffraction planes (002), (100), and (101). Also, increasing the temperature improves the peak intensity of the SiC of all constituents of the nanocomposite to reach values of 141, 8658, 733, 420, 652, 1092, 1121 cps of Fe_3_O_4_, SiO_2_, CNT, Si, Graphite, 6H-SiC, and 3C-SiC, respectively, at 1000 °C^77,78^.

To reduce any amorphous SiO_2_ in SiC–SiO_2_ composite product soaking in 1 M NaOH followed by 5% HF treatment to reduce the ratio of crystalline SiO_2_ and increase the SiC ratio. So accordingly, all characteristic peaks are maximized except the peaks of SiO_2_ intensity and integration were decreased (Fig. 5C).

### TEM and SEM analysis of different construction of (CAPA)–SiC composite

**Figure 6:**
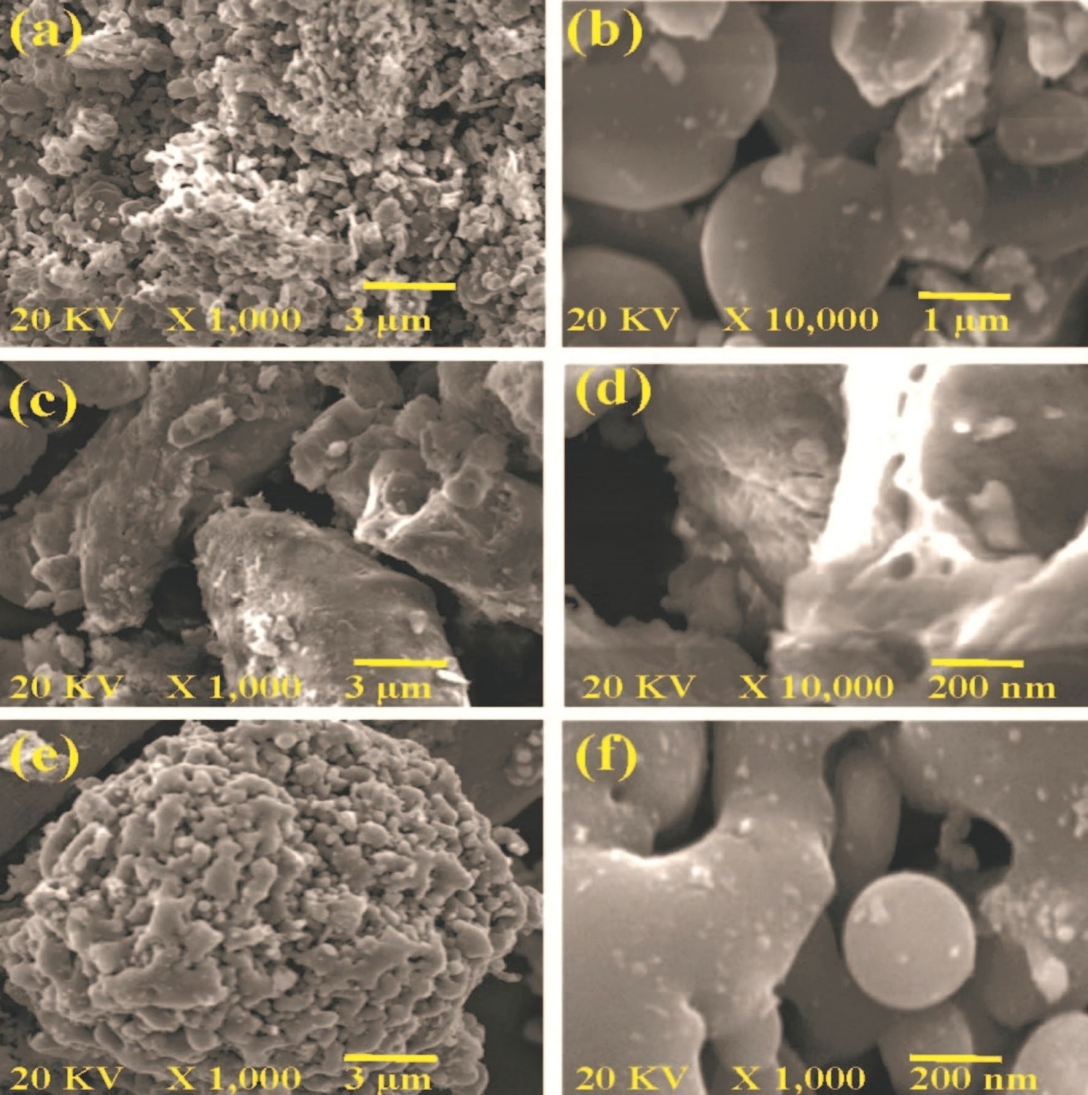
SEM of (**a**, **b**) marine sponge, (**c**, **d**) SiC–SiO_2_ nanocomposite (**e**, **f**) SiC–SiO_2_ nanocomposite after soaking in HF.

The magnifications of the first composite of SiC–SiO_2_ give no appearance of a nanotube (Fig. 6a,b), but there is a sudden appearance of the nanotubes after treatment with 5% HF. This appearance of nanotube is related to the decrease in SiO_2_ amount that allows the SiC nanotube to appear (Fig. 6c,d). After the addition of SiC to the polymer chain of CAPA, the polymer circulates SiC giving a compact structure of polymerized nanostructure/microstructure (Fig. 6e,f).

**Figure 7:**
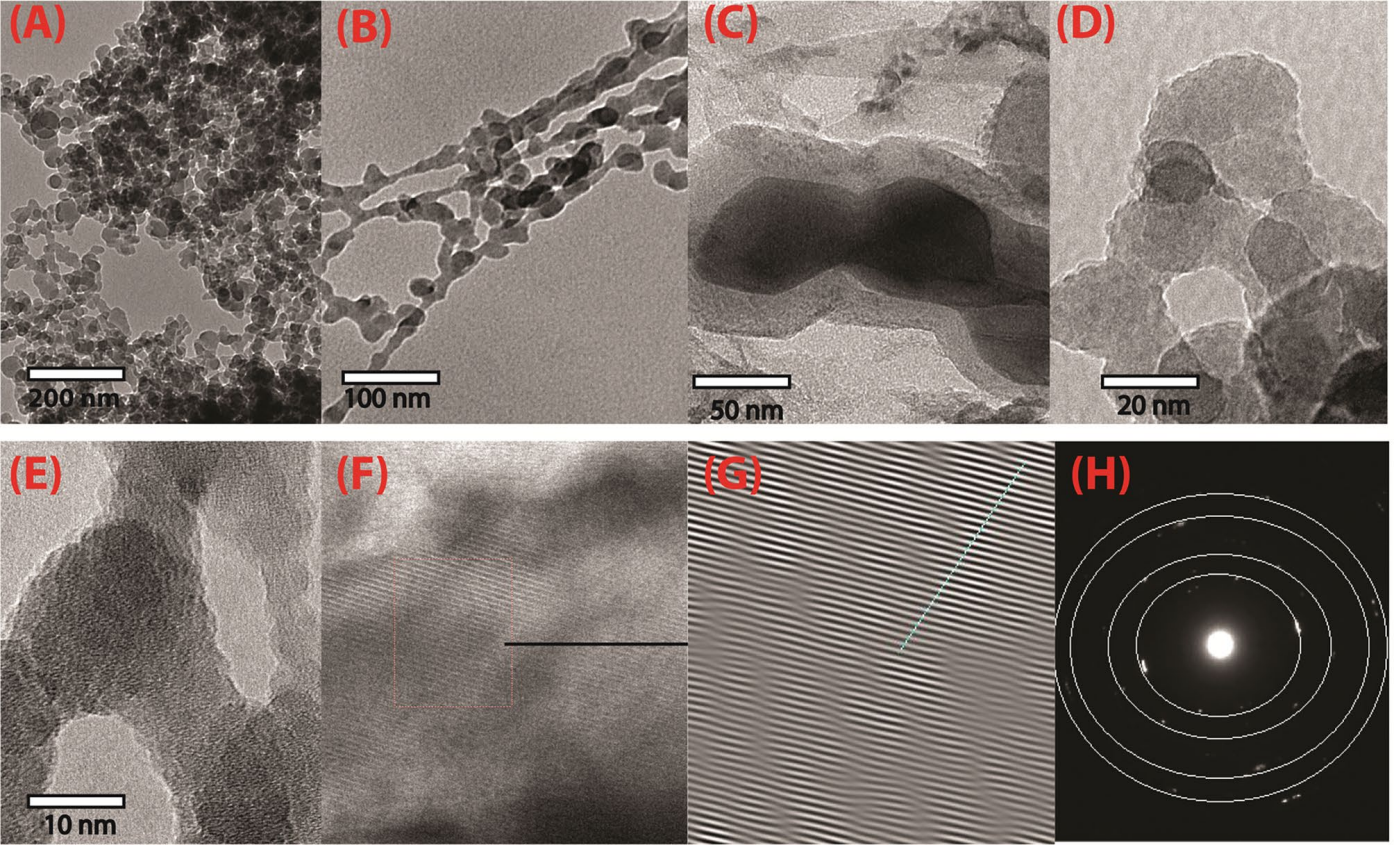
TEM (CAPA)-SiC. (**A**–**F**), TEM images HRTEM of 3C-SiC (**G**, **H**).

The outer morphology of the pyrolysis product is similar to the skeleton branches of a width of about 10 nm, indicating a slow rate of crystal growth (Fig. 7A–E). Investigation of the innermost cover marked by the development of SiC coat verified through the HRTEM image without crystalline building and the SAED pattern without clear diffraction rings (Fig. 7F–H). SiC–SiO_2_ composite is almost fully converted into crystals of large size, attended by the presence of a bright and sharp SiC (111) ring with some discontinuous spots (Fig. 7H).

### Corrosion protection estimation

**Figure 8:**
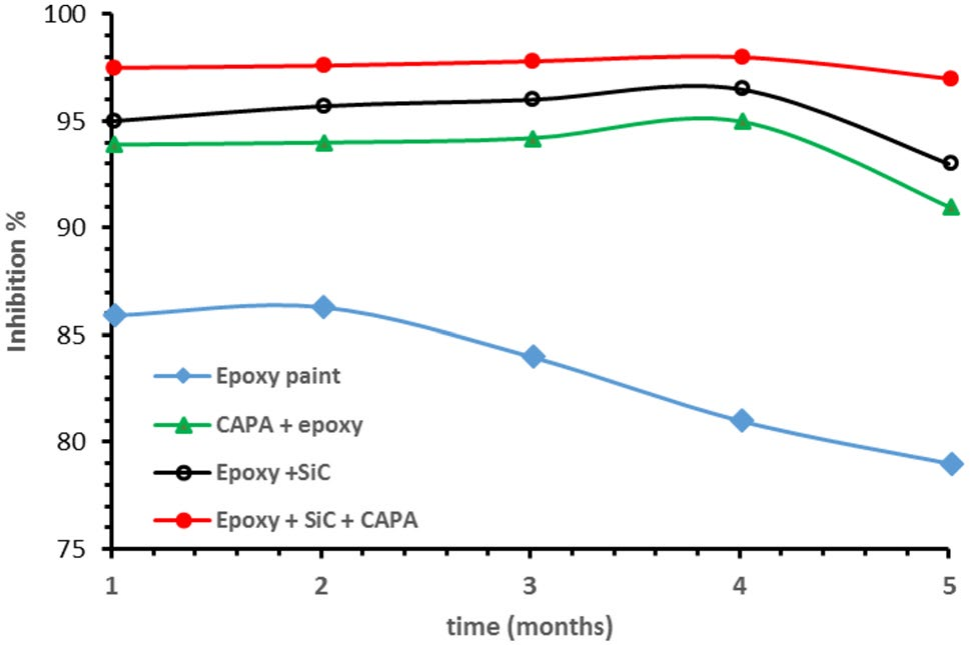
Inhibition efficiency in the presence of different coating types in a 3.5 wt% NaCl solution resulting from weight loss experiments performed on copper substrate.

Weight loss tests were conducted at different interval times to evaluate the time required for the coatings to decrease their optimum efficiency and stability. Table 2 and Fig. 8 present the corresponding values of inhibition efficiency and corrosion resistance of copper. The data shows that the corrosion inhibition efficiency showed almost constant percent during 5 months in the case of (SiC–epoxy) and (CAPA–epoxy) and starts to decrease after 4 months. After 5 months of testing period, the inhibition efficiency was achieved and nearly constant. The lowest rate of inhibition and poor behavior was in the case of epoxy only, which was damaged after 1 month.

**Table 2:** Dependency of time-based coating effectiveness and corrosion resistance via weight loss measurements obtained on a copper in a 3.5 wt% NaCl solution in the presence of different coating samples.

Coating	CR g/cm^2^. year					Time/*IE*%					Time/θ				
	1	2	3	4	5	1	2	3	4	5	1	2	3	4	5
Epoxy	1.37	0.66	0.51	0.46	0.51	85.9	86.3	84.0	81.0	79.0	0.86	0.86	0.84	0.81	0.79
CAPA–epoxy	0.59	0.29	0.19	0.12	0.21	39.9	94.0	94.2	95.0	91.0	0.40	0.94	0.94	0.95	0.91
Epoxy–SiC	0.48	0.21	0.13	0.08	0.17	95.0	95.7	96.0	96.5	93.0	0.95	0.96	0.96	0.96	0.93
Epoxy–CAPA–SiC	0.24	0.11	0.07	0.04	0.07	97.5	97.6	97.8	98.0	97.0	0.97	0.98	0.98	0.98	0.97

The corrosion rate CR (g/cm^2^. year) of metal in the case of the gravimetric method was Calculated from the following Eq. (4):

4 $${\text{CR}} = \frac{{\Delta {\text{m}}}}{{{\text{At}}}}{*}365$$

where Δm is the weight difference in gram (g), A, is specimen surface area (cm^2^) and t is exposed corrosion time (day). The corrosion rate of the specimens of non-coated and coated copper immersed in a solution of 3.5 wt% NaCl was determined as a function of immersion time at 30 °C. The corrosion rates obtained (CR) are shown in Table 2. The corrosion rate of copper decreases with time. From 2 months of immersion, the corrosion rate stabilizes with time. This is due to a protective film that forms on the surface of the copper. The corrosion rate of copper decreases through different coating layers under all periods of immersion times to be minimum in Epoxy–CAPA–SiC after 5 months.

**Figure 9:**
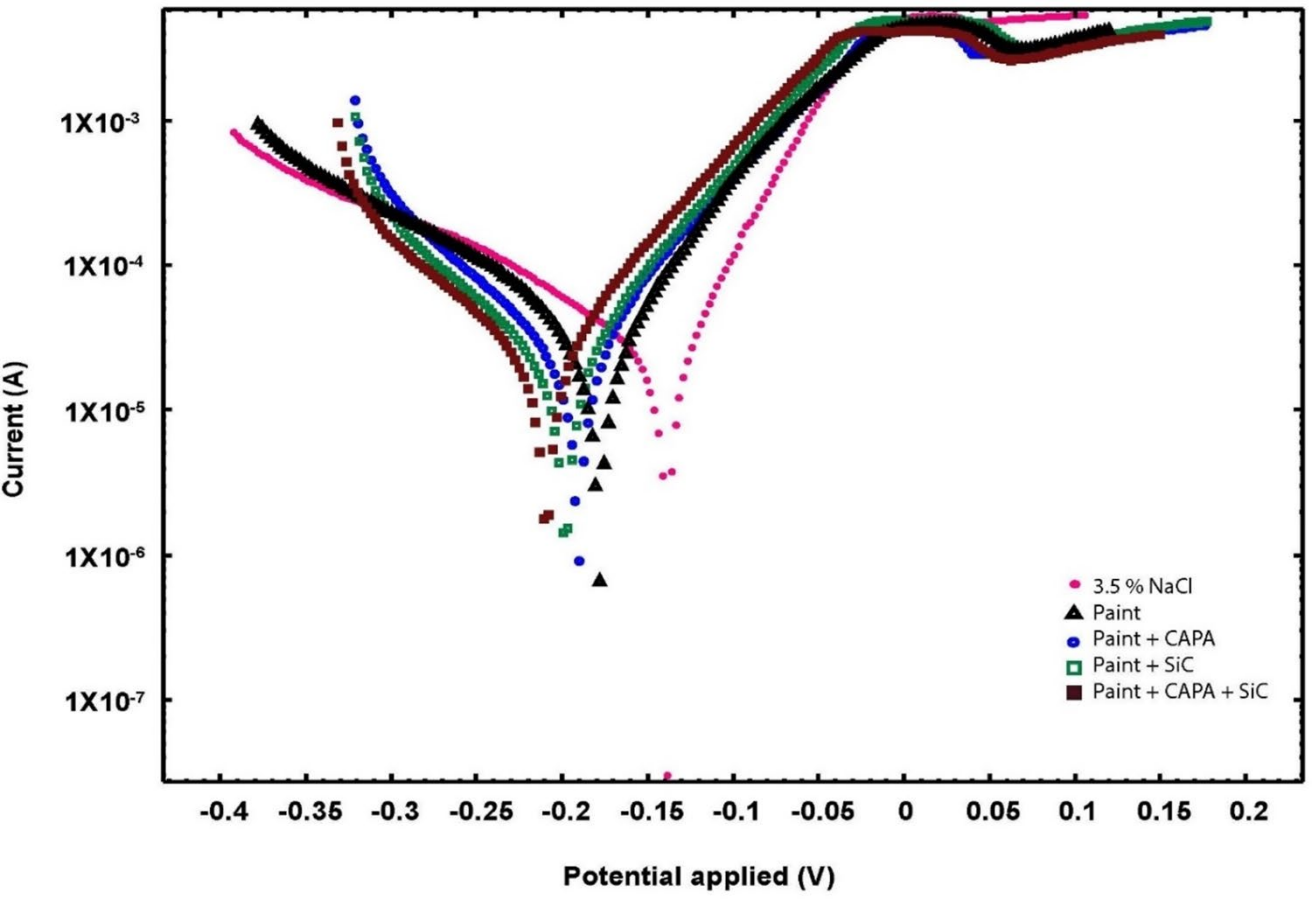
Tafel plots of different copper samples treated with 3.5 wt% NaCl solution.

One of the most effective techniques for evaluating a copper coating’s capacity against corrosion is Tafel polarization plots. The good corrosion resistance of coatings is typically related to a minor corrosion rate (*C*_R_), which equates to a greater corrosion potential (*E*_corr_) or a lower corrosion current density in a typical Tafel polarization curve (*i*_corr_)^79^. As shown in Fig. 9, after immersion of the copper in 3.5 wt% NaCl without a covering layer, the potential is recorded at negative values, which indicates more corrosion tendency of copper in 3.5 wt% NaCl. All coatings tafel plots were shifted to more negative values and this shift is greater in (paint + SiC + CAPA), which means thermodynamically the corrosion tendency in the presence of coats is lower due to cathodic protection. βa and βc have small variations in data that indicate that the studied samples have similar mechanisms in the corrosion process.

Important information is also presented in Fig. 9, including Tafel plots of coated and uncoated copper samples submerged in a 3.5% wt NaCl solution. The anodic and cathodic curves make it simple to identify the Tafel regions, and Table 3 lists the corrosion data that were calculated using the Tafel extrapolation way. Equation (5) was used to determine the CR values of the coated and uncoated copper samples.

5 $$C_{R } = \frac{{M \left( {\text{g}} \right) \times I_{corr} \left( {{\text{A}}\;{\text{cm}}^{ - 1 } } \right)}}{{n \times P \left( {{\text{g}}\;{\text{cm}}^{ - 1} } \right)}} \times 3270$$

where *M* is the molecular mass of copper, i_corr_ is calculated by an intersection of the linear region of the cathodic and anodic divisions of the Tafel curves, *n* is the number of electrons that are lost during the oxidation reaction (valence), and the density of copper (*p*). From Table 3 and Eq. (5), we can notice that the corrosion data of all coated copper samples are better than that of uncoated copper. Important information is also presented in Fig. 8, including Tafel plots of coated and uncoated copper samples submerged in a 3.5 wt% NaCl solution. 3 distinct regions of Tafel were noticed; the active dissolution region (apparent Tafel region), the active-to-passive transition region, and the limiting current region. The anodic polarization curve in copper demonstrated a monotonic rise in current with potential up until the current reached its maximum value. Following this peak current density, the current density rapidly decreased as the potential increased, creating a narrow anodic current peak that was connected to the formation of the CuCl film. The passivation peak area increases until it reaches its maximum at (paint + SiC + CAPA), which is associated with the passivation film’s nonhomogeneity.be readily recognized as the anodic.

**Table 3:** Fitting corrosion information for copper tasters dipped in 3.5 wt % NaCl solution using Tafel polarization measurement.

Samples	− *E*_corr_ (mV)	*i*_corr_ (µA cm^−2^)	*C*_R_ mm/year	*β*a (mV dec^−1^)	*β*c (mV dec^−1^)	*PE* (%)
Uncoated copper	155	17.17	0.19	39	72	0.0
paint coating	180	2.48	0.03	32	68	86.0
Paint + CAPA + coating	190	1.06	0.012	46	69	94.3
Paint + Si + coating	205	0.78	0.009	48	66	95.6
Paint + CAPA + Si coating	220	0.53	0.0006	49	68	97.4

**Figure 10:**
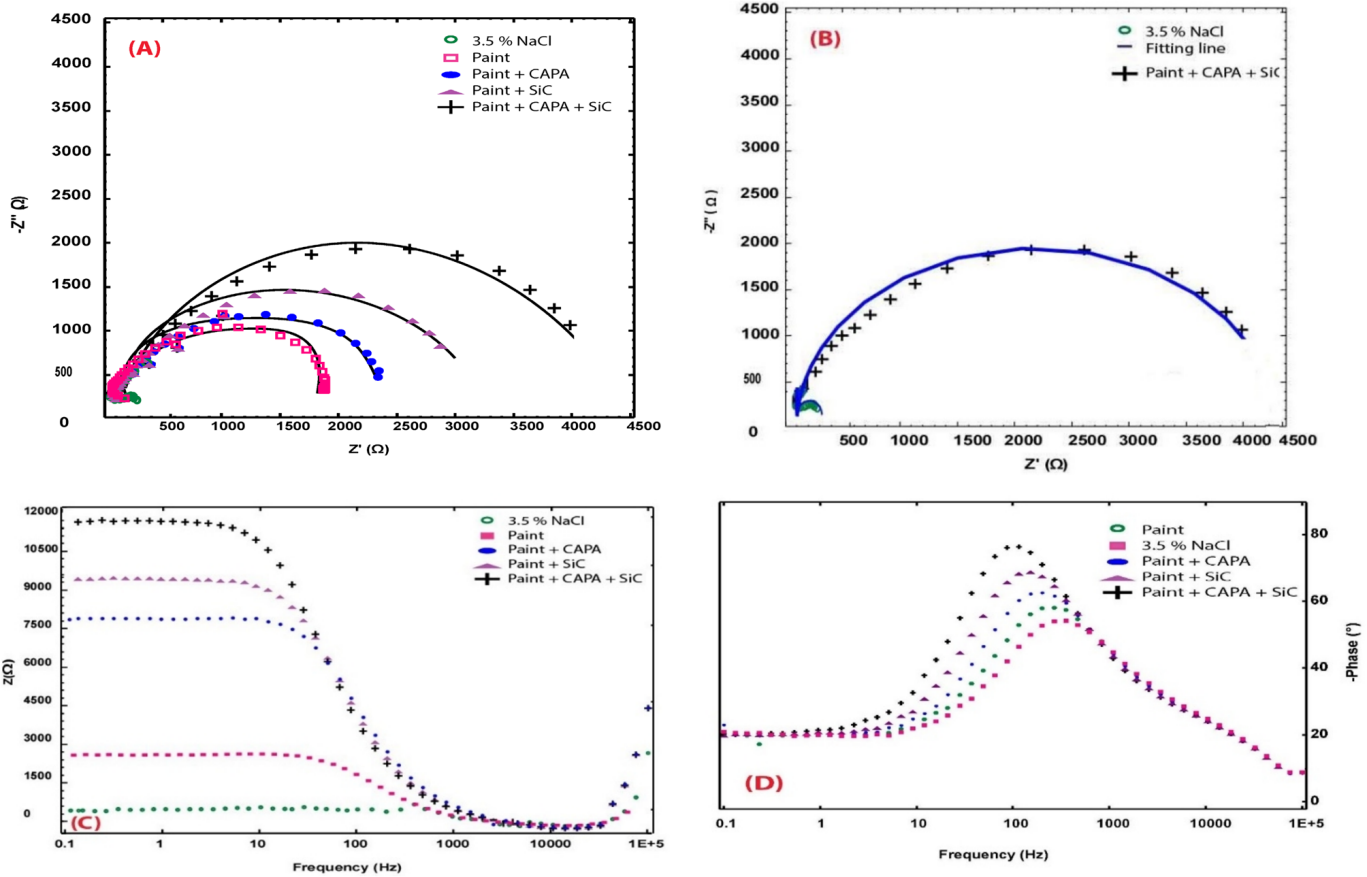
(**A**) Nyquist plots (**B**, **C**) Bode plot, (**D**) Ceta plot of uncoated and coated copper coupons dipped in 3.5 wt% NaCl solution.

**Figure 11:**
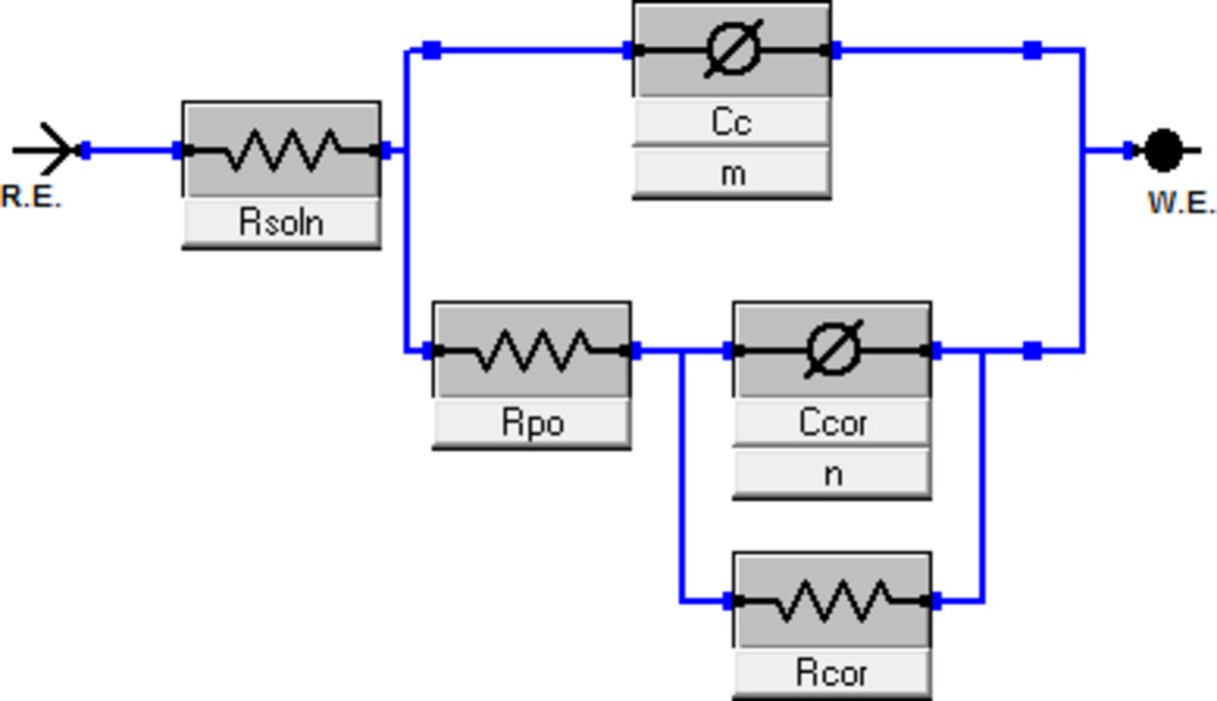
Fitting model of impedance curves of Blanc and coated copper samples immersed in 3.5 wt% NaCl solution.

EIS in a 3.5 wt% NaCl solution is another electrochemical technique for examining the corrosion behaviors of coated and uncoated copper. Nyquist plots of coated and blank copper submerged in a 3.5 wt% NaCl solution is displayed in Fig. 10A. The copper impedance diagrams in 3.5 wt% NaCl resembles those in the literature in terms of shape (Table 4)^80^. Coatings raise impedance but do not affect the other aspects of the corrosion mechanism that are caused by their presence. The measured data is represented by symbols in Fig. 10, and the fitting data acquired by using the equivalent circuit (Fig. 11) is represented by solid lines^81^. Table 3 lists the parameters that were determined by fitting the experimental data with the equivalent circuit as well as the estimated inhibition efficiencies. The Nyquist plots shown in Fig. 10A show that there is little difference in the characters of the plots for coated and uncoated copper electrodes.

**Table 4:** Fitting of the impedance parameters for copper samples immersed in 3.5 wt% NaCl solution by *EIS* measurements.

Samples	R_s_ (ohm)	R_cor_ (ohm)	R_po_ (ohm)	Q_f_		Q_dl_		Goodness	PE (%)
				*C*_cor_ (µF cm^−2^)	n	*C*_c_ (µF cm^−2^ )	n	× 10^−3^	
Uncoated copper	3.782	89	350	12.35	0.956	129	0.782	4.1	0
paint coating	3.598	129	2800	8.71	0.989	70.82	0.679	4.9	87.5
Paint + CAPA + coating	4.120	124	7100	7.59	0.897	61.57	0.754	3.8	95.0
Paint + Si–C + coating	2.986	136	9200	6.23	0.991	55.10	0.781	6.4	96.1
Paint + CAPA + Si–C coating	2.978	145	12,000	5.51	1.000	41.98	0.668	4.6	97.0

**Figure 12:**
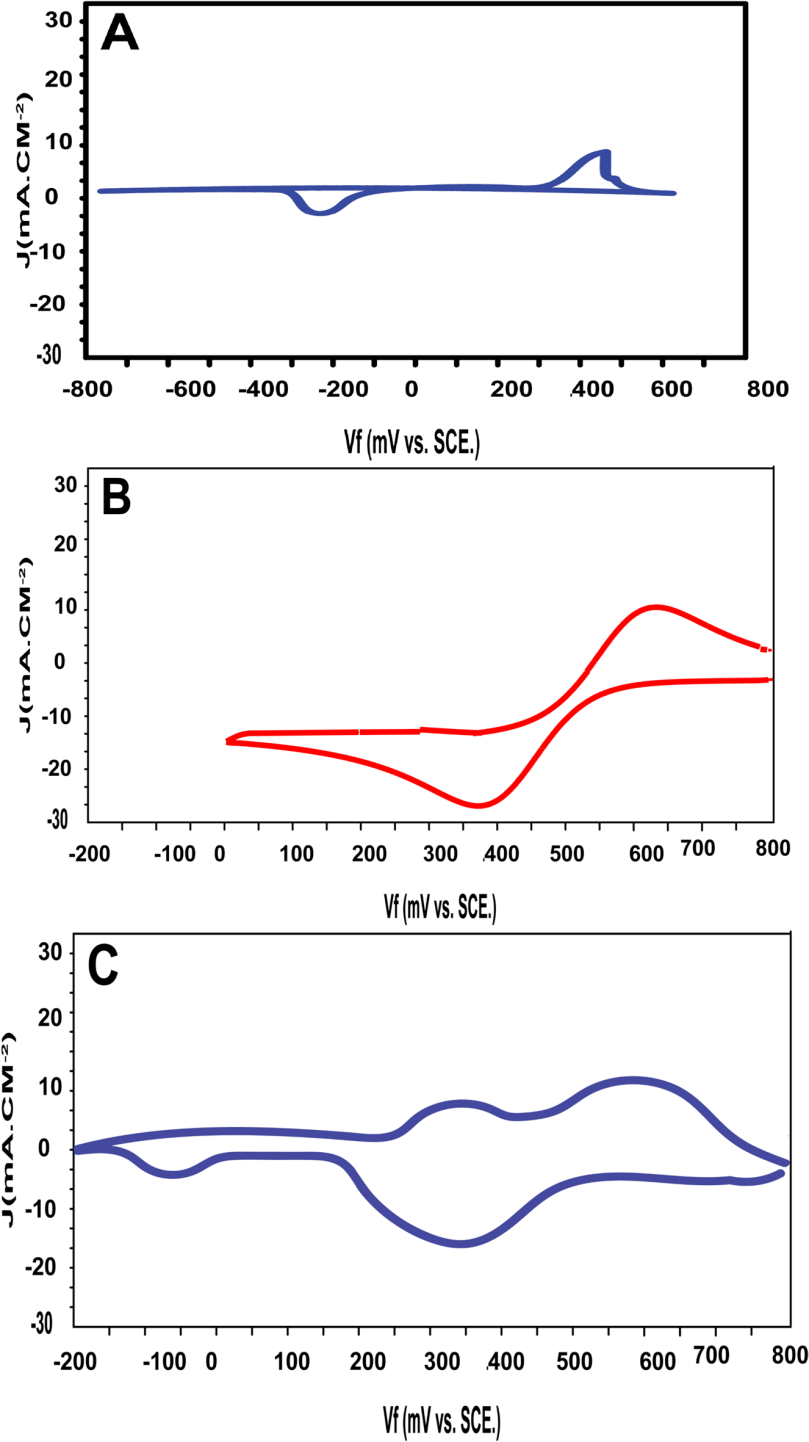
Cyclic voltammograms of (**A**) copper, (**B**) SiC, (**C**) CA-SiC/copper in 3.5 wt% NaCl.

The electrochemical behaviour of copper in 3.5 wt% NaCl solution was studied by cyclic voltammetry. It can be seen that although the voltammograms present several anodic peaks there is always only one cathodic peak. Both anodic peaks represent the formation of CuCl_2_, Fig. 12A. This behaviour can be explained according to the well-accepted mechanism of copper dissolution in chloride media. According to this mechanism, the first anodic peak is due to the formation of the CuCl precipitate on the electrode surface. This layer is porous and an electrochemical reaction can continue through it and a second anodic peak is observed. The second anodic peak is followed by a current plateau. The intensity of this plateau points to the existence of a poorly protective film.

The electrochemical behaviour of SiC precipitated on graphite electrode in 3.5 wt% NaCl solution was studied by cyclic voltammetry. It can be seen that although the voltammograms present one anodic peak and one cathodic peak, Fig. 12B. The electrochemical behaviour of CA-SiC precipitated on a copper electrode in 3.5 wt% NaCl solution was studied by cyclic voltammetry. The graph certified still presence of SiC on the surface of copper after treatment in 3.5 wt% NaCl during 5 months, Fig. 12C.

### Structure, morphology, and effect of dipping time of coatings on the surface of copper

**Figure 13:**
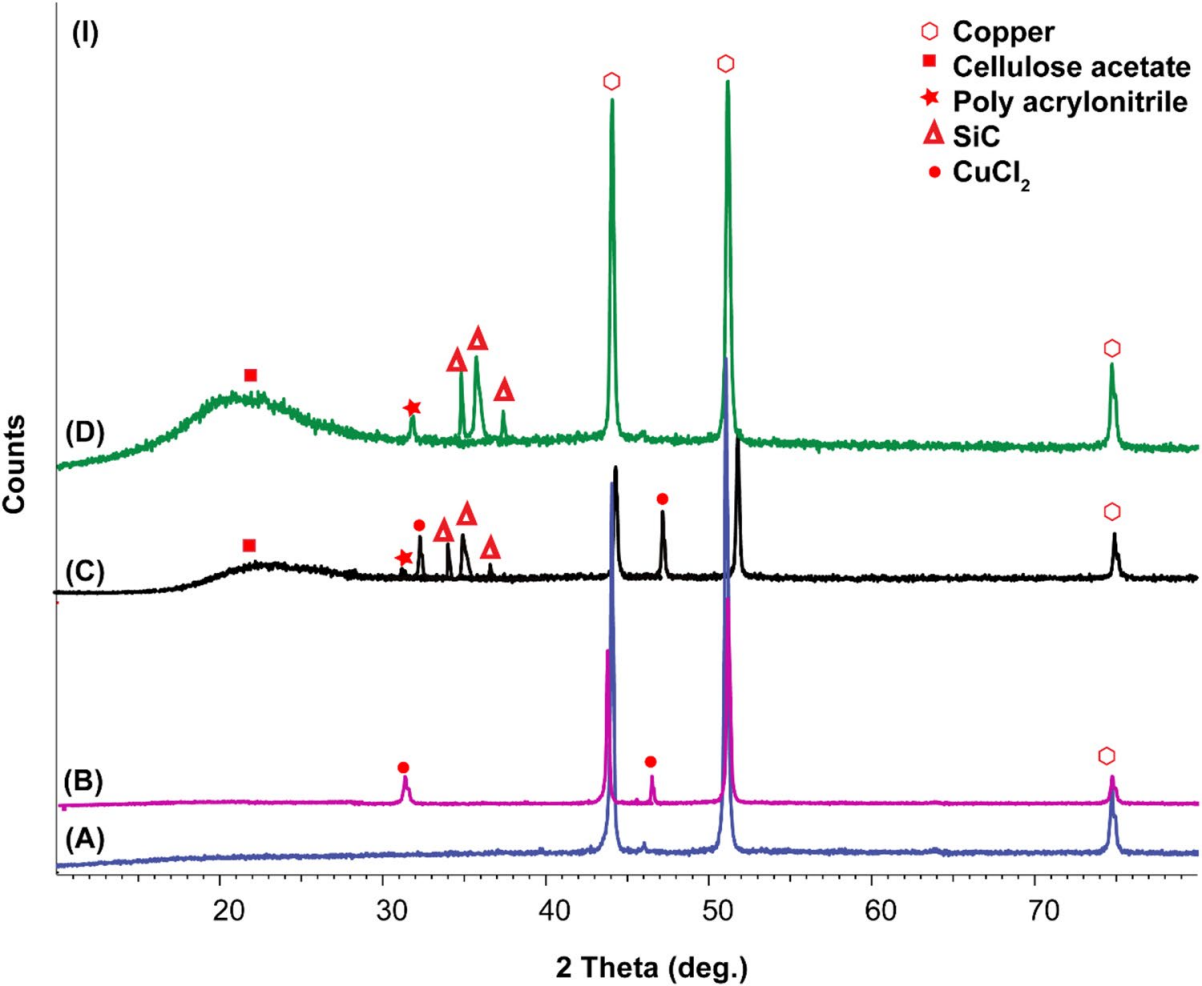
X-ray diffraction patterns after washing of (**A**) copper (**B**) copper + 3.5 wt% NaCl (**C**) copper + CAPA–SiC + epoxy, (**D**) copper + CAPA–SiC + epoxy 3.5 wt% NaCl.

The effect of 3.5 wt% NaCl solution as a simulation of seawater on the corrosion of copper and its inhibition using different forms of epoxy and SiC coating is important to be studied after a long period of immersion time due to the slow corrosion mechanism of copper (Fig. 13A–D). The XRD before and after 4 months of immersion time shows that CAPA + Si–C with epoxy coating has almost no variation in type and intensity of peaks as discussed through the following charts.

Copper displays diffraction peaks at about 43.8°, 50.8°, and 74.4°, which were attributed to typical peaks of copper. After immersion in 3.5 wt% NaCl, new characteristic peaks at 31° and 47° that correspond to CuCl_2_ (Fig. 13A,B)^78^. Other diffraction peaks were displayed at 20°, which were attributed to cellulose acetate and 30° corresponds to acrylonitrile, 35.6, 36° and 37° corresponds to SiC (Fig. 13D,C)^82^. All previous peaks have almost less change in intensity than before immersion in 3.5 wt% NaCl with only the appearance of peaks at 31° and 47° that correspond to CuCl_2_ (Fig. 13A,B).

**Figure 14:**
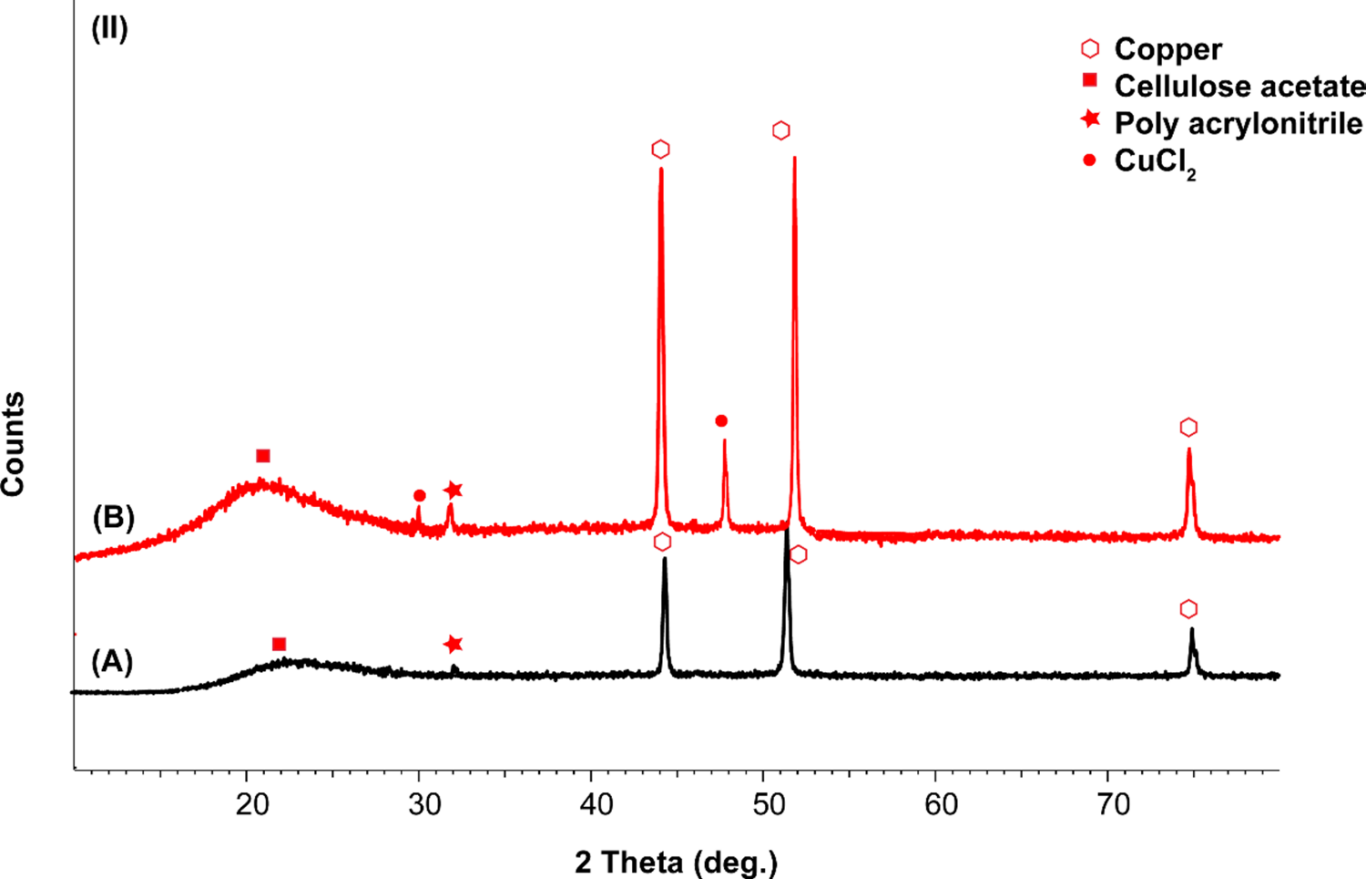
X-ray diffraction patterns after washing of (**A**) copper, (**B**) copper + CAPA–epoxy + 3.5 wt% NaCl.

CAPA without SiC is also studied as a coating on copper before and after immersion in 3.5 wt% NaCl. XRD shows that characteristic peaks of copper have more intensity than before corrosion with the appearance of CuCl_2_. This may be related to the amount of CAPA minimized by the effect of corrosion and the copper surface became exposed to corrosive solution and X-ray through analysis (Fig. 14A,B).

**Figure 15:**
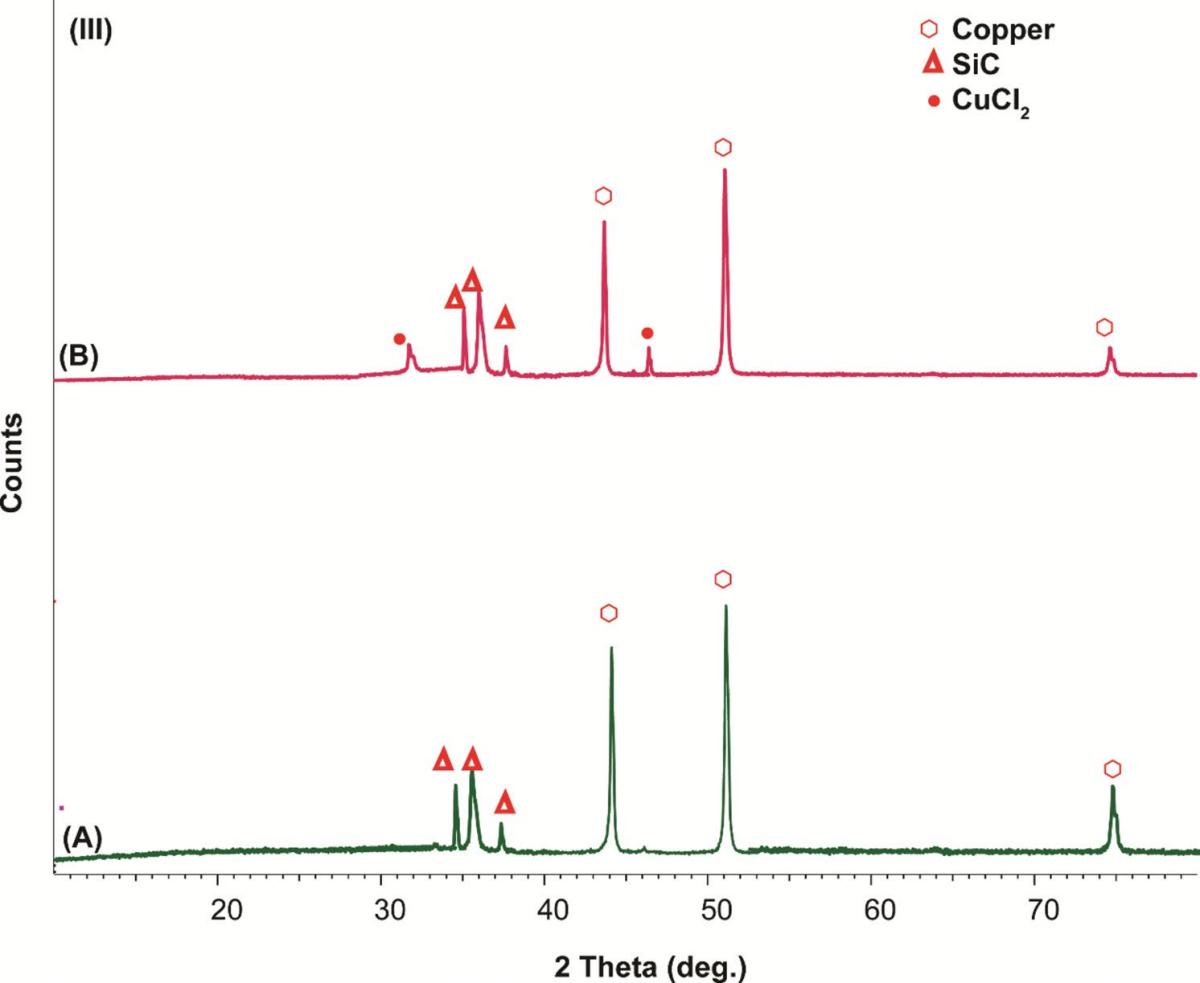
X-ray diffraction patterns after washing of (**A**) copper + 3.5 wt% NaCl, (**B**) copper + SiC–epoxy + 3.5 wt% NaCl.

Another important factor is to study the influence of SiC with epoxy only on the corrosion of copper. The peaks before and after corrosion have new peaks of CuCl_2_ and noticeable no change were displayed at 20°, which were attributed to cellulose acetate and 30° corresponding to acrylonitrile, 35.6, 36° and 37° corresponding to SiC. All previous peaks have almost less change in intensity than before immersion in 3.5 wt% NaCl with the significant appearance of peaks at 31° and 47° that correspond to CuCl_2_ (Fig. 15A,B).

### Impact of time on the surface morphology and efficiency of different coating types

**Figure 16:**
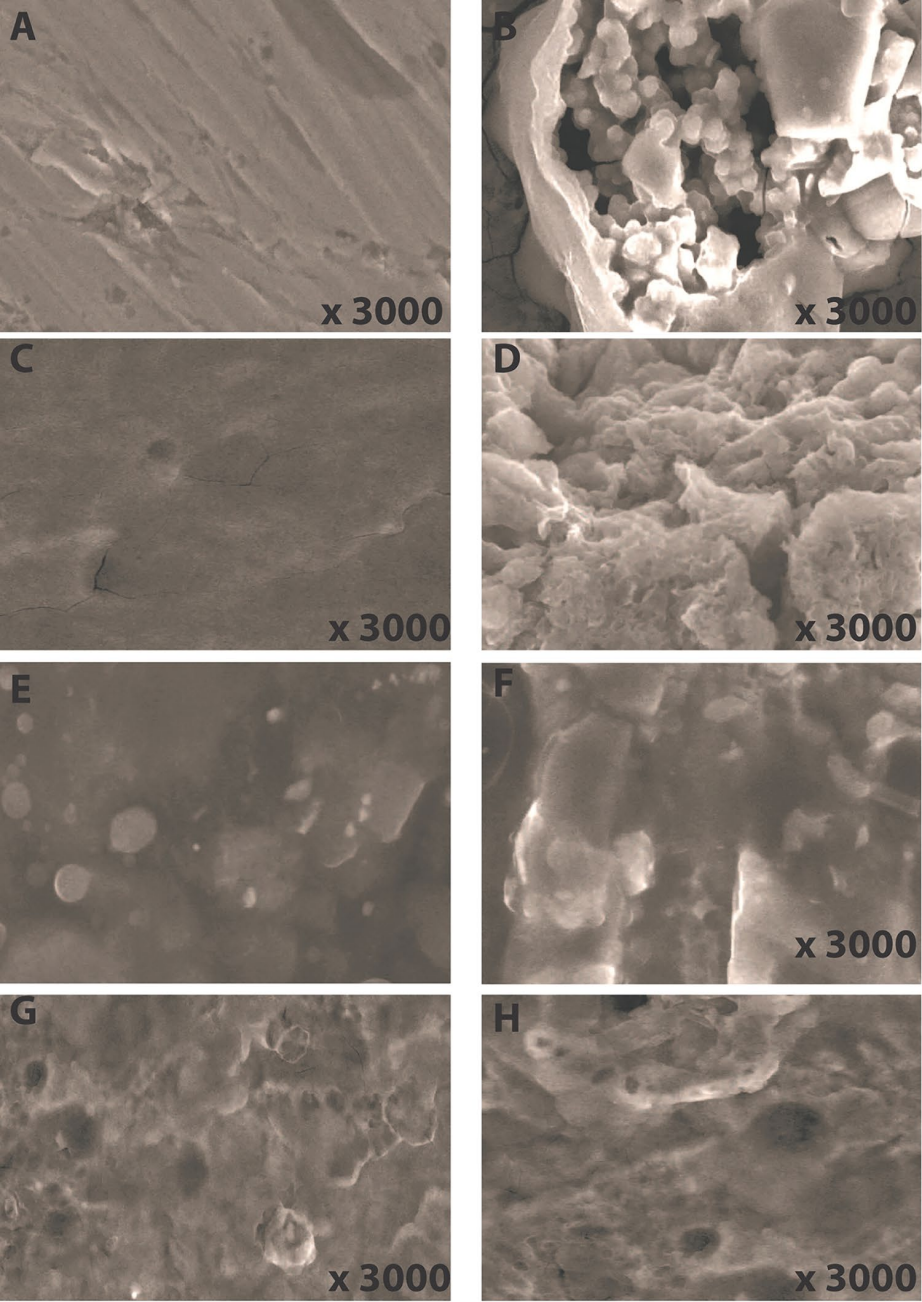
SEM analysis of different coating types before (**A**, **C**, **E**, **G**: copper, epoxy–CAPA, epoxy–SiC, Epoxy–SiC–CAPA) and after immersion (**B**, **D**, **F**, **H**), respectively.

The external of different copper samples was immersed in 3.5 wt% NaCl. After 5 months the samples were washed following the drying steps. Scanning electron microscopy was applied to obtain morphological information about the surface of copper (Fig. 16). The results displayed in Fig. 16A,B showed aggressive behavior of NaCl on the surface of the copper for 5 months. This behavior starts to be reduced in acceptable percent in the case of epoxy coating with CAPA (Fig. 16C,D) and more improvement in the case of SiC with epoxy (Fig. 16E,F) to arrive maximum excellent behavior in the case of SiC–CAPA with epoxy, (Fig. 16G,H).

## Suggested mechanism of corrosion protection

**Figure 17:**
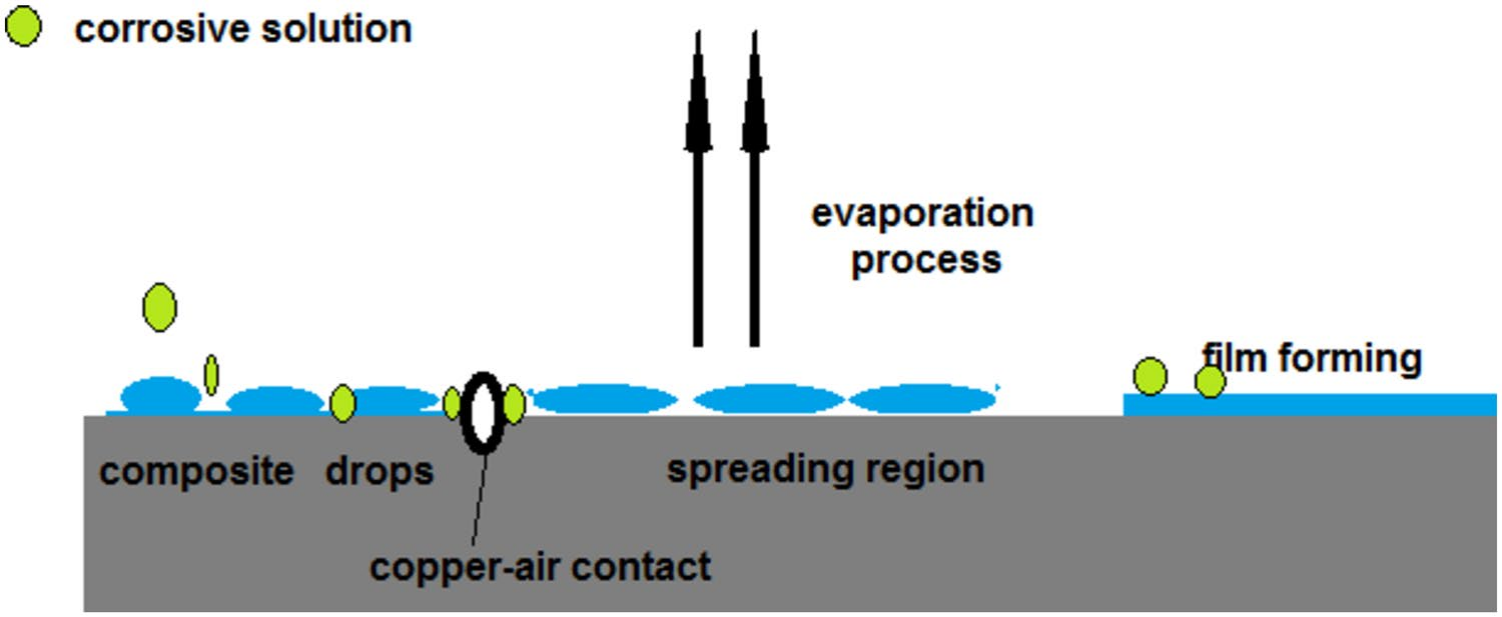
Expected mechanism of film formation on a copper surface.

The proposed mechanism of film formation on the copper surface using CAPA–SiC is presented in Fig. 17. From BET calculations, the surface area of epoxy paint is higher than CAPA–SiC-paint composite. But the pore volume is higher than those of the other samples. However, the pore volume CAPA–SiC-paint composite decreases through the induction of SiC which is related to being SiC filler into the polymer. The pore volume is important factor in the treatment of corrosion. After film formation, approximately no copper-air contact regions that were approved from BET and SEM measurements.

## Conclusions

The produced CAPA–SiC composite was prepared by chemical oxidative polymerization. The composite has good attachment to the copper surface via mixing with commercial epoxy paint using a casting technique The surface area of CAPA–SiC composite copolymers mixed with epoxy paint is much higher than those of the other samples. The SiC decreases pore volume giving an excellent protective layer on copper. . By using Tafel polarization and electrochemical impedance spectroscopy tests in a 3.5 wt% NaCl solution, the inhibition efficiency increased from 86 to 79% in the presence of CAPA–SiC/epoxy paint coating.

## Data Availability

The datasets used in this investigation are accessible for review upon request from the corresponding author of the paper.
